# Assessing the contrast sensitivity function in myopic parafovea: A quick contrast sensitivity functions study

**DOI:** 10.3389/fnins.2022.971009

**Published:** 2022-10-06

**Authors:** Zixuan Xu, Yijing Zhuang, Zhipeng Chen, Fang Hou, Lily Y. L. Chan, Lei Feng, Qingqing Ye, Yunsi He, Yusong Zhou, Yu Jia, Junpeng Yuan, Zhong-Lin Lu, Jinrong Li

**Affiliations:** ^1^State Key Laboratory of Ophthalmology, Zhongshan Ophthalmic Center, Sun Yat-sen University, Guangzhou, China; ^2^School of Ophthalmology and Optometry and Eye Hospital, Wenzhou Medical University, Wenzhou, Zhejiang, China; ^3^School of Optometry, The Hong Kong Polytechnic University, Kowloon, Hong Kong SAR, China; ^4^Division of Arts and Sciences, New York University (NYU) Shanghai, Shanghai, China; ^5^Center for Neural Science and Department of Psychology, New York University, New York, NY, United States; ^6^New York University-East China Normal University (NYU-ECNU) Institute of Brain and Cognitive Neuroscience, Shanghai, China

**Keywords:** contrast sensitivity function (CSF), peripheral visual field, quick CSF, myopia, parafovea

## Abstract

**Purpose:**

Compare peripheral contrast sensitivity functions (CSF) between myopes and emmetropes to reveal potential myogenic risks during emmetropization.

**Materials and methods:**

This observational, cross-sectional, non-consecutive case study included data from 19 myopes (23.42 ± 4.03 years old) and 12 emmetropes (22.93 ± 2.91 years old) who underwent central and peripheral quick CSF (qCSF) measurements. Summary CSF metrics including the cut-off spatial frequency (cut-off SF), area under log CSF (AULCSF), low-, intermediate-, and high-spatial-frequency AULCSFs (l-, i-, and h-SF AULCSFs), and log CS at 19 SFs in the fovea and 15 peripheral locations (superior, inferior, temporal, and nasal quadrants at 6, 12, 18, and 24° eccentricities, excluding the physiological scotoma at 18°) were analyzed with 3-way and 4-way between-subjects analysis of variance (ANOVA) (α = 0.05).

**Results:**

Three-way ANOVA showed that myopes had significantly increased AULCSF at 6° (mean difference, 0.08; 95% CI, 0.02–0.13; *P* = 0.007) and 12° (mean difference, 0.09; 95% CI, 0.03–0.14; *P* = 0.003). Log CS at all 19 SFs were higher in the myopia group compared to the normal group (mean differencesuperior, 0.02; 95% CI, 0.01–0.20; *P* = 0.02 and mean differenceinferior, 0.11; 95% CI, 0.02–0.21; *P* = 0.01) at 12°. The h-SF AULCSF at 6° (mean differenceinferior, 1.27; 95% CI, 0.32–2.22; *P* = 0.009) and i-SF AULCSF at 12° (mean differencesuperior, 5.31; 95% CI, 4.35–6.27; *P* < 0.001; mean differenceinferior, 1.14; 95% CI, 0.19–2.10; *P* = 0.02) were higher in myopia vs. normal group.

**Conclusion:**

We found myopia increased contrast sensitivity in superior and inferior visual field locations at 6° parafoveal and 12° perifoveal regions of the retina. The observation of increased contrast sensitivities within the macula visual field in myopia might provide important insights for myopia control during emmetropization.

## Introduction

Myopia has long been a serious public health and economic concern affecting a significant proportion of the population worldwide (Lam et al., 2011). Over the last two decades, animal research on peripheral defocus has generated important insights on its effects in regulating eye growth related to myopia development (Smith et al., 2007). As such, there is good consensus that both central and peripheral visual signals can interfere with the emmetropization mechanism (Liu and Wildsoet, 2011). Many studies highlight how optical factors impact structural changes related to the peripheral retina. There are very few studies on the contrast sensitivity function to better understand the real-world influence of spatial vision on myopia, and its effects on perceptual response to peripheral defocus (blur).

Studies on how emmetropization is tuned to particular spatial frequencies are of interest because visual images on the retina are composed of different spatial frequencies, which may, in turn, regulate eye growth during emmetropization (Smith et al., 2005). From the perspective of environmental visual experience, it is thought that visual inputs to the retina associated with indoor activities such as reading, writing and use of electronic devices contain much less out-of-focus signals compared to those of variable focal planes in outdoor activities (Flitcroft, 2012; Muriel et al., 2013). During most indoor activities, peripheral vision is filled with high contrast, well-focused images from the same focal plane. We hypothesized that myopia may affect peripheral contrast sensitivity functions. As a first attempt to explore this hypothesis, we assessed central and peripheral contrast sensitivity functions (CSF) using the quick CSF (qCSF) method in myopia and normal vision.

Compared to visual acuity (VA), which only assesses spatial resolution at high contrast, CSF can provide a more comprehensive assessment of spatial vision, including its optical, retinal, neural, and adaptation abilities (Arden, 1978). The conventional laboratory psychophysical methods have limited application for clinical practice because of their long test times (Lesmes et al., 2010). Preprinted letter or grating CSF charts are imprecise because of their coarse sampling of spatial frequency and contrast (Hou et al., 2016). The qCSF method was developed to measure CSF based on the Bayesian adaptive test framework (Lesmes et al., 2010; Lu and Dosher, 2013; Michael et al., 2013). Using an active learning algorithm, it can precisely and accurately measure a CSF curve in 3–5 min (Lesmes et al., 2010; Zheng et al., 2019). More recently, the Bayesian adaptive testing framework has extended to measure light sensitivity (Xu et al., 2019) and contrast sensitivity (Rosen et al., 2014; Xu et al., 2020) visual field maps. However, the novel psychophysics paradigms have not been applied to assess peripheral spatial vision across the full spectrum of spatial frequencies in myopia.

In this study, we seek to evaluate and compare central and peripheral CSFs between myopes and emmetropes. The results might yield potential insights for myopia prevention, diagnostic workup, and management from the perspective of peripheral spatial vision modulation.

## Materials and methods

### Subjects and inclusion criteria

This was an observational, cross-sectional, non-consecutive case study. Eligible healthy volunteers were recruited from The Optometry Clinic of Zhongshan Ophthalmic Center, Guangzhou, China. Ethics approval was obtained from the Zhongshan Ophthalmic Center Ethics Committee. All participants provided informed consent after receiving both written and verbal explanations of the nature and intent of the study.

Healthy volunteers between 18 and 35 years old, with no history of surgery or ocular disease, normal binocular vision and accommodative function, no physical/mental health histories, and a natural pupil diameter of 4–6 mm, were recruited for this study. Inclusion criteria included: emmetropia (less than −0.50D to +1.00D) and/or low-to-moderate myopia (−0.50 to −6.00 D inclusive), refractive astigmatism no greater than −0.50 D cylinder, with corrected-to-normal visual acuities of 0.00 logMAR or better. All recruited participants had normal accommodation and convergence function, and wore their full optical correction during all the tests. Axial length (AL) was measured three times with IOL-Master 500 (Carl Zeiss Meditec, Ag, Jena, Germany). The examinations were performed by the same specialized technician, and the average value was recorded. Soft contact lenses were used for optical correction because they provided relatively natural peripheral vision, while spectacles may induce distortions and compromise peripheral optical quality (Patino et al., 2010). All tests were conducted monocularly with the fellow eye occluded.

### Objective visual quality examination

Objective evaluation of the optical quality of each participant’s visual system was performed using the double-pass Optical Quality Analysis System II (OQAS II, Visiometrics, Terrassa, Spain). The system generates several metrics, including the Modulation Transfer Function (MTF) cut-off frequency, Strehl ratio, OQAS Values, and the Objective Scattering Index (OSI) (an objective parameter to describe intraocular light scatter). The MTF cut-off frequency represents the spatial frequency (cycles per degree, cpd) at which the MTF reaches 0.01. The Strehl ratio represents the ratio of peak focal intensity between the aberrated and ideal point spread functions. There are three OQAS Values (OVs), OV100, OV20, and OV9%, corresponding to the spatial frequencies of the modulation transfer function at 100, 20, and 9% contrasts.

### Central and peripheral quick contrast sensitivity functions measurements

**FIGURE 1:**
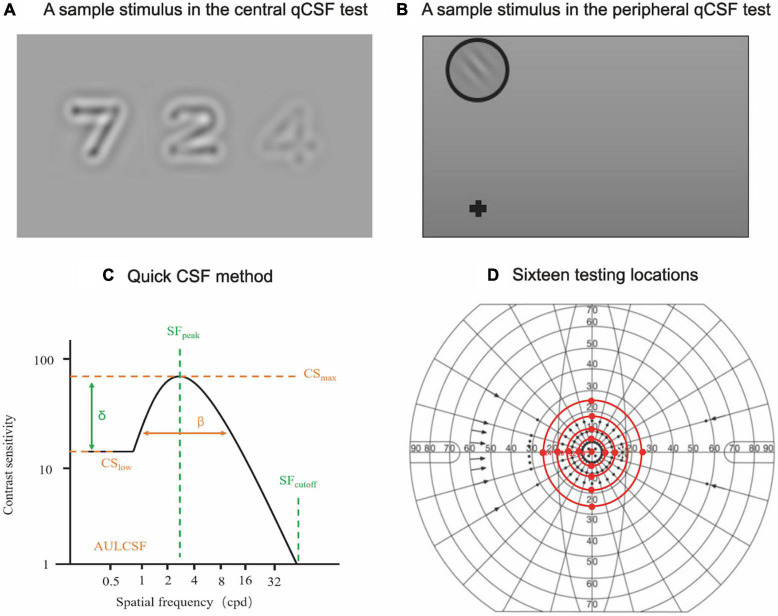
Central and peripheral qCSF measurements. **(A)** A sample stimulus depicting digits presented on the LCD monitor during the central qCSF test. **(B)** A sample stimulus presented on the LCD monitor for the peripheral qCSF test. **(C)** The truncated log-parabola CSF model used in the qCSF paradigm, with four parameters: peak sensitivity (CSmax), peak SF (SFpeak), bandwidth (β), and low-SF truncation (δ) (Barbot et al., 2021). **(D)** Sixteen testing locations (central and peripheral testing locations, excluding the physiological scotoma) in the peripheral CSF test.

The digit qCSF method was used to assess CSF at the fovea in a dark room, at a 4 m test distance. Details of the qCSF method have been previously reported (Dorr et al., 2015). Briefly, the stimuli were presented on a gamma-corrected 46-inch LCD monitor (Model: NEC LCD P463), with a 1920 × 1080 pixel resolution, mean luminance of 90 cd/m^2^, and a vertical refresh rate of 60 Hz. In each trial, participants were asked to verbally report the digits presented on the screen to the examiner, who used a computer keyboard to enter the responses (Figure 1A). The stimuli disappeared after all responses were entered. Observers were given an option to report (I don’t know), and the response was regarded as incorrect. No feedback was provided during the test. A new trial began 500 ms later. The procedure used a 10-alternative forced-choice (AFC) digit identification task to measure CSF in 35 trials. The process took approximately 5 min.

Contrast sensitivity functions in peripheral vision was measured by the grating qCSF method with a 2-AFC grating orientation identification task. Stimuli were displayed at four adjusted test distances (3.08, 1.96, 1.43, and 1.12 m for tests at 6, 12, 18, and 24° eccentricities, respectively) to measure CSF at fifteen peripheral locations (superior, inferior, temporal, and nasal quadrants at 6, 12, 18, and 24° eccentricities, excluding the physiological scotoma at 18°). Participants were required to maintain foveal fixation at all times. The grating stimuli were presented every 50 ms with different frequencies and contrasts. In each trial, participants were asked to verbally report the grating orientation (Figure 1B). There were 160 trials in each test that took approximately 5 min. A complete peripheral qCSF measurement (6, 12, 18, and 24°) for two eyes took approximately 90 min (Figure 1D). Participants were given two to three peripheral qCSF practice trials to familiarize themselves with the test requirements before formal data collection, with a 1-min rest period between tests.

The 19 log spatial frequencies (log SF) were different at different eccentricities (Supplementary Table 1). The area under log CSF (AULCSF), was calculated as the area under the log CSF curve from 0.5 to 18 cpd. The Cut-off spatial frequency (Cut-off SF) was defined as the SF at which CS is 2.0 (threshold = 0.5) (Yan et al., 2017). The cut-off SF, AULCSF, and log CS at 19 SFs (equally spaced in log units) at each of the 16 testing locations were derived from the estimated CSF curve (Figure 1C; Hou et al., 2016; Barbot et al., 2021). The log CS value was plotted as a function of log SF, where second-order polynomials were fitted to the data. The best-fitting functions were integrated from 0.70 to 3.00 cpd, from 3.10 to 12.00 cpd, and from 12.10 to 20.00 cpd to derive low (l), intermediate (i), and high (h) spatial-frequencies (l-, i-, and h-SF) AULCSFs.

### Statistical analysis

Data normality was assessed by the Kolmogorov-Smirnov test, and are presented as means ± standard deviation (SD). The *t*-test was used to explore the differences in age, AL, MTF cut-off, Strehl ratio, OVs at 100, 20, and 9% contrasts, and OSI between myopia and normal groups. A three-way between-subjects analysis of variance (ANOVA) was performed to evaluate the between-subjects effects of group (myopia and normal), location (superior, nasal, inferior, temporal) and eccentricity (central, 6, 12, 18, and 24°) as well as their interactions on AULCSF and cut-off SF. A four-way between-subjects ANOVA was performed to evaluate the between-subjects effects of group (myopia and normal), location (superior, nasal, inferior, temporal), eccentricity (6, 12, 18, and 24°) and SF range (low, intermediate, and high) as well as their interactions on l-, i-, and h-SF AULCSFs. Another four-way between-subjects ANOVA was performed to evaluate the between-subjects effects of group (myopia and normal), location (superior, nasal, inferior, temporal), eccentricity (6, 12, 18, and 24°) and SF (19 values) as well as their interactions on logCS. *Post-hoc* Bonferroni correction was used for pairwise comparisons. If the homogeneity of variance assumption is not satisfied, Brown-Forsythe correction was used to treat the heterogeneity between groups. Because the temporal 18° location was close to the physiological scotoma, its AULCSF, cut-off SF and l-, i-, and h-SF AULCSFs were based on the average of the adjacent points in the statistical analysis. A *p*-value of < 0.05 was considered to be statistically significant. All statistical analyses were conducted using SPSS version 19 (SPSS, Chicago, IL, USA).

## Results

### Demographic and objective optical quality assessments

The demographic and clinical characteristics of the participants are listed in Table 1. Of the 19 subjects who were corrected to 0.00 logMAR or better, 32 eyes with low to moderate myopia and 20 eyes from 12 emmetropes were tested in this study. Six subjects in the myopia group and four subjects in the emmetropia group were only tested monocularly because their other eye had refractive errors which did not meet the inclusion criteria. Other than a significant difference in mean spherical equivalent refraction and AL (**P = 0.001, *t*-test), there was no significant difference in gender, age and mean spherical equivalent refractivebest corrected visual acuity (BCVA) between the normal and myopia groups (all *P* > 0.10, *t*-test). There was also no significant difference between the two groups in their optical quality assessment (Table 2; all *P* > 0.10, *t*-test).

**TABLE 1:** Clinical characteristics of the participants.

Characteristic	Myopia group *n* = 19 (32 eyes)	Normal group *n* = 12 (20 eyes)	*p*-value
Male (%)	10 (52)	6 (50)	1.00
Age (years), mean (SD)	23.42 (4.03)	22.93 (2.91)	0.86
Spherical Equivalent Refraction (D), mean (SD)	–3.02 (1.60)	–0.34 (0.52)	0.001**
[–3D, –0.5D] (*n* = 20), mean (SD)	–2.00 (0.63)		
[–6D, –3D] (*n* = 12), mean (SD)	–4.68 (0.73)		
Best Corrected Visual Acuity (logMAR), mean (SD)	–0.10 (0.10)	–0.10 (0.10)	0.98
Axial length (mm)	24.87 (0.44)	23.76 (0.40)	0.001**

SD, standard deviation. **Statistically significant difference between myopia group and normal group (*t*-test, **p = 0.001).

**TABLE 2:** Optical quality measurements.

OQAS parameter	Myopia group	Normal group	*p*-value
MTF cutoff, mean (SD) (95% CI)	41.72 (15.58) (30.29–55.05)	44.84 (15.80) (32.72–57.09)	0.85
Strehl ratio, mean (SD) (95% CI)	0.23 (0.12) (0.15–0.37)	0.26 (0.14) (0.16–0.42)	0.67
OV 100%, mean (SD) (95% CI)	1.38 (0.52) (1.00–1.80)	1.47 (0.48) (1.10–1.90)	0.61
OV 20%, mean (SD) (95% CI)	0.99 (0.52) (0.60–1.60)	1.12 (0.48) (0.80–1.70)	0.39
OV 9%, mean (SD) (95% CI)	0.63 (0.38) (0.40–1.10)	0.64 (0.52) (0.40–1.20)	0.97
OSI, mean (SD) (95% CI)	0.53 (0.56) (0.20–1.20)	0.42 (0.88) (0.10–1.90)	0.64

OQAS, Optical Quality Assessment System; SD, standard deviation; CI, Confidence Interval.

### Contrast sensitivity functions metrics

**FIGURE 2:**
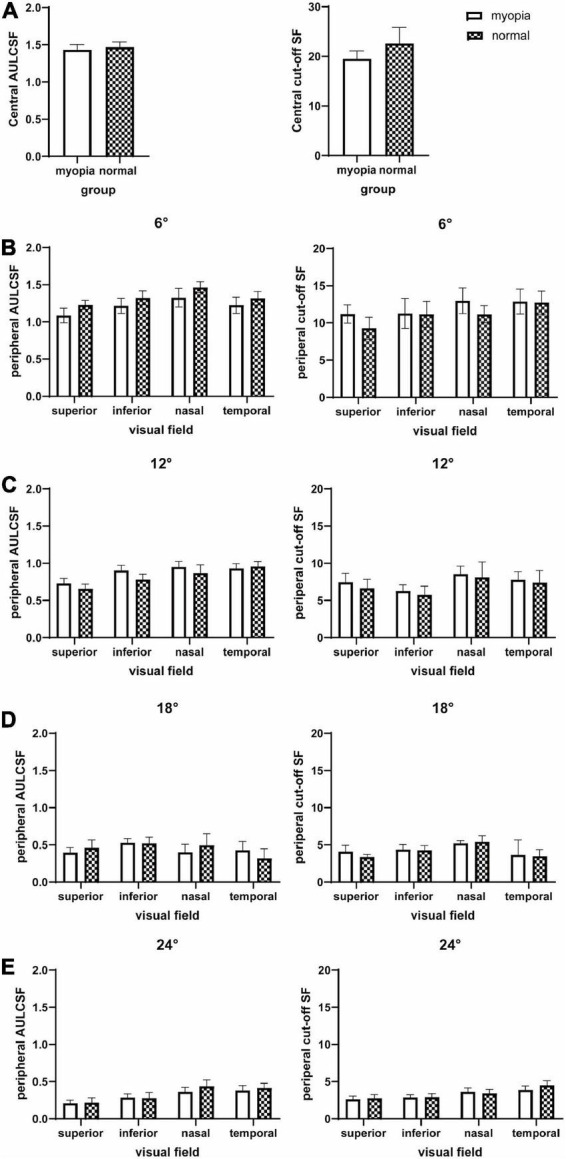
AULCSF and cut-off SF at the fovea and 16 peripheral locations in the visual field. **(A)** Central, **(B)** peripheral 6°, **(C)** peripheral 12°, **(D)** peripheral 18°, and **(E)** peripheral 24°. The plot shows the mean and 95% confidence limits. The white columns denote the myopia group and the shaded columns denote the normal group.

**FIGURE 3:**
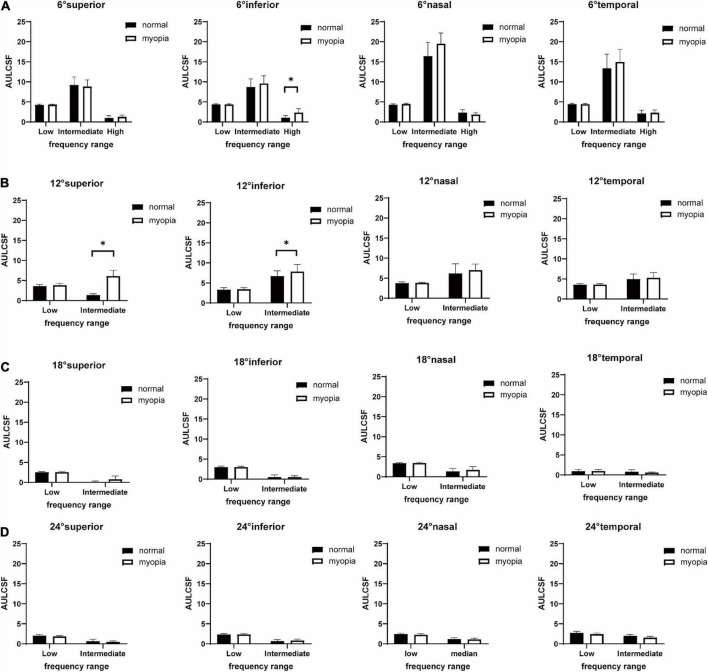
Histographs of low-, intermediate-, and high-SF AULCSFs (Mean ± SD) of myopes and emmetropes at superior, inferior, nasal, temporal visual fields locations at **(A)** peripheral 6°, **(B)** peripheral 12°, **(C)** peripheral 18°, **(D)** peripheral 24°. The plot shows the mean and 95% confidence limits. The white columns denote the myopia group and the black columns denote the normal group. Significant differences (*P < 0.05, *Post-hoc* Bonferroni test) between myopes and emmetropes are indicated by a (*).

**FIGURE 4:**
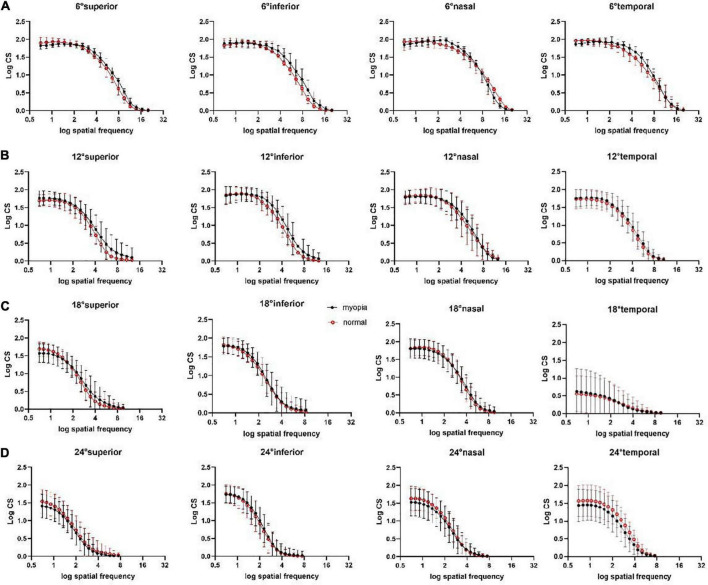
Peripheral CSFs at superior, inferior, nasal, temporal visual field locations at **(A)** peripheral 6°, **(B)** peripheral 12°, **(C)** peripheral 18°, **(D)** peripheral 24°. Each curve represents the fit of second-order polynomials to the average observed CSF. The middle points of the vertical line segments denote the mean log CS values; the top and bottom of the vertical line segments represent the 25th and 75th percentile values.

The mean and SD of AULCSF, cut-off SF and l-, i-, and h-SF AULCSFs at all 16 test locations for the two groups are presented in Table 3 and Figures 2, 3. CSFs at all 16 test locations for the two groups are shown in Figure 4.

**TABLE 3:** Summary of CSF metrics.

		Myopia group				Normal group			
			
	Location	Superior	Nasal	Inferior	Temporal	Superior	Nasal	Inferior	Temporal
AULCSF, mean (SD)	Central	1.433 (0.176)				1.468 (0.983)			
	6°	1.085 (0.270)	1.325 (0.330)	1.216 (0.284)	1.224 (0.300)	1.227 (0.170)	1.464 (0.188)	1.323 (0.261)	1.318 (0.250)
	12°	0.794 (0.276)	0.997 (0.196)	0.935 (0.250)	0.930 (0.173)	0.670 (0.139)	0.870 (0.234)	0.795 (0.151)	0.985 (0.134)
	18°	0.396 (0.182)	0.401 (0.294)	0.532 (0.183^)^	0.428 (0.326)*	0.458 (0.232^)^	0.494 (0.327)	0.527 (0.153)	0.318 (0.279)*
	24°	0.208 (0.124)	0.335 (0.187)	0.288 (0.125)	0.380 (0.187)	0.218 (0.135)	0.358 (0.126)	0.281 (0.157)	0.415 (0.134)
Cut-off SF, mean (SD)	Central	19.496 (4.137)				22.590 (4.523)			
	6°	11.510 (2.677)	12.980 (2.691)	11.268 (5.197)	12.996 (3.371)	9.292 (2.870)	11.164 (3.260)	11.177 (3.366)	12.996 (3.371)
	12°	7.442 (3.063)	8.548 (2.713)	6.273 (1.949)	7.291 (1.724)	6.648 (2.063)	8.106 (3.284)	5.740 (2.149)	7.0.47 (1.576)
	18°	4.084 (1.608)	5.212 (0.885)	4.376 (1.644)	3.663 (1.917)*	3.353 (0.558)	5.408 (1.387)	4.278 (1.160)	3.471 (1.071)*
	24°	2.810 (0.837)	3.531 (1.101)	3.314 (1.504)	3.838 (1.048)	2.923 (1.100)	3.584 (0.936)	2.951 (0.968)	4.413 (1.116)
l-SF AULCSF, mean (SD)	6°	4.315 (0.322)	4.489 (0.534)	4.349 (0.434)	4.421 (0.508)	4.261 (0.341)	4.311 (0.414)	4.355 (0.377)	4.453 (0.429)
	12°	3.844 (0.451)	3.822 (0.467)	3.449 (0.426)	3.593 (0.654)	3.603 (0.410)	3.356 (0.486)^†^ (^†^*P* = 0.03)	3.315 (0.507)	3.556 (0.504)
	18°	2.552 (0.633)	3.464 (0.505)^†^ (^†^*P* < 0.001)	3.008 (0.561)^†^ (^†^*P* = 0.01)	0.842 (0.849)^†^* (^†^*P* = 0.01)	3.356 (0.486)	3.434 (0.527)^†^ (^†^*P* < 0.001)	3.018 (0.468)^†^ (^†^*P* = 0.03)	0.934 (0.958)^†^* (^†^*P* < 0.001)
	24°	1.855 (0.460)	2.281 (0.753)^†^ (^†^*P* = 0.02)	2.237 (0.524) ^†^ (^†^*P* = 0.002)	2.424 (0.815)^†^ (^†^*P* = 0.001)	2.069 (0.586)	2.457 (0.489)	2.343 (0.492)	2.783 (0.784)^†^ (^†^*P* < 0.001)
i-SF AULCSF, mean (SD)	6°	8.841 (4.264)	19.519 (6.493) ^†^ (^†^*P* < 0.001)	9.608 (4.129) ^†^ (^†^*P* < 0.001)	14.967 (7.803) ^†^ (^†^*P* < 0.001)	9.229 (3.448)	16.421 (4.752)	8.719 (3.801)	13.355 (7.209)
	12°	6.683 (1.457)	7.889 (1.428)	7.859 (1.765)	7.223 (1.764)	1.374 (0.782)^‡^ (^‡^*P* < 0.001)	7.650 (1.989)^†^ (^†^*P* = 0.002)	6.716 (1.311) ^†‡^ (^†^*P* = 0.012) (^‡^*P* = 0.02)	6.950 (1.378)^†^ (^†^*p* = 0.007)
	18°	0.781 (2.244)	1.747 (2.208)	0.526 (0.845)	0.851 (0.916)*	0.017 (0.714)	1.359 (1.551)	0.506 (1.162)	0.912 (1.281)*
	24°	0.467 (0.559)	1.099 (0.913)	0.839 (0.946)	1.581 (0.924)	0.686 (0.969)	1.213 (0.680)	0.706 (0.783)	2.031 (0.764)
h-SF AULCSF mean (SD)	6°	1.272 (1.224)	1.837 (1.233)	2.340 (2.653)	2.328 (1.830)	1.002 (1.163)	2.349 (1.509)	1.071 (1.123)^‡^ (^‡^*P* = 0.009)	2.111 (1.758)

(i) *The physiological scotoma.

(ii) ^†^Statistically significant difference between different locations at the same eccentricities within the group (*post-hoc* Bonferroni test, ^†^*p* < 0.05).

(iii) ^‡^Statistically significant difference between different groups at the same eccentricities and locations (*post-hoc* Bonferroni test, ^‡^*p* < 0.05).

#### Area under log contrast sensitivity function and cut-off SF

A three-way between-subjects ANOVA on AULCSF revealed that location (**P < 0.001), eccentricity (**P < 0.001), and location × eccentricity (**P < 0.001) and eccentricity × group (*P = 0.01) interactions were significant (Supplementary Table 2). *Post-hoc* Bonferroni tests highlight four significant differences: (1) for both groups, the peripheral locations at the superior qudrant showed a lower AULCSF than the nasal and temporal AULCSFs at 6° (*P < 0.05); (2) for both groups, there were significant AULCSF differences between all pairs of equal-eccentricity locations at 12, 18, 24° (*P < 0.05), except between the inferior and temporal locations at 12° and inferior and superior/nasal locations at 24° (*P* > 0.10); (3) for both groups, there were significant AULCSF differences between every two adjacent eccentricities at all locations (**P < 0.001); and (4) the AULCSF of the myopia group was higher than that of the normal group in all locations (central, superior, nasal, inferior, temporal) at 6° (*post-hoc* Bonferroni test, mean difference, 0.08; 95% CI, 0.02–0.13; *P = 0.007) and 12° (*post-hoc* Bonferroni test, mean difference, 0.09; 95% CI, 0.03–0.14; *P = 0.003), but there was no significant AULCSF difference between the two groups at 18° and 24° (*P* > 0.10).

A three-way between-subjects ANOVA on Cut-off SF revealed that location (**P < 0.001), eccentricity (**P < 0.001), and location × eccentricity interaction (**P < 0.001) were significant (Supplementary Table 2). *Post-hoc* Bonferroni tests found that, (1) for both groups, the superior Cutoff SF was lower than the nasal Cutoff SF at 6 and 12°, and temporal Cut-off SF at 6° (*P < 0.05). There was no significant Cut-off SF difference between any other equal-eccentricity locations at other eccentricities (*P* > 0.10); (2) for both groups, there were significant Cut-off SF differences between every two adjacent eccentricities at all locations (*P < 0.05), and (3) there was no significant Cut-off SF difference between the myopia and normal groups (*P* > 0.10).

#### Low-, intermediate-, and high-SF area under log contrast sensitivity functions

The results of four-way between-subjects ANOVA on l- and i-SF AULCSFs are shown in Supplementary Table 3. There are significant effects of location (**P < 0.001), eccentricity (**P < 0.001), group (*P = 0.02), SF range (**P < 0.001), location × eccentricity (**P < 0.001), location × SF range (**P < 0.001), eccentricity × SF range (**P < 0.001), group × SF range (*P = 0.02), location × eccentricity × group (*P = 0.009), location × eccentricity × SF range (**P < 0.001), and location × eccentricity × group × SF range (*P = 0.009). Further analysis showed that, for l-SF AULCSF, the location × eccentricity × group interaction was not significant (*P* = 0.96). However, for i-SF AULCSF, the location × eccentricity × group interaction was significant (*P = 0.009), and the location × group interaction was only significant at 12° (**P < 0.001).

*Post-hoc* analyses indicated that (1) in the myopia group, there were significant l-SF AULCSF differences between the superior and nasal/inferior/temporal visual field locations at 18° and 24° (*P < 0.05), and significant i-SF AULCSF differences between the superior and nasal/inferior/temporal visual field locations at 6° (**P < 0.001); (2) in the normal group, there were significant l-SF AULCSF differences between superior and nasal/inferior visual field locations at 18° (*P < 0.05), and significant i-SF AULCSF differences between the superior and nasal/inferior/temporal visual field locations at 12° (*P < 0.05); (3) for both groups, there were significant l-SF AULCSF differences between two adjacent eccentricities at all locations (**P < 0.001), but there was no significant i-SF AULCSF difference between 18 and 24° at any location in the myopia group (*P* > 0.10), and between 12, 18, and 24° at the superior/inferior/nasal locations and between 18 and 24° at the temporal location in the normal group (*P* > 0.10); and (4) the i-SF AULCSF of the myopia group was higher than that of normal group at the superior (mean difference, 5.31; 95% CI, 4.35–6.27; **P < 0.001) and inferior (mean difference, 1.14; 95% CI, 0.19–2.10; *P = 0.02) visual field locations at 12°.

Because CSFs at 12, 18, and 24°cuts off at i-SF, h-SF AULCSF was only computed at 6° visual field locations. A two-way ANOVA on h-SF AULCSF (Supplementary Table 4) showed a significant effect on location (*P = 0.007). *Post-hoc* analyses showed that (1) there was no significant h-SF AULCSF difference between any two equal-eccentricity locations in the two groups (*P* > 0.10); and (2) the h-SF AULCSF of the myopia group was higher than that of the normal group at the inferior visual field location at 6° (mean difference, 1.27; 95% CI, 0.32–2.22; *P = 0.009).

#### Contrast sensitivities

Second-order polynomials were fitted to measured CSFs at all visual field locations (Figure 4). The results of four-way between-subjects ANOVA on log CS (Supplementary Table 5) revealed that location, eccentricity, group, SF, location × eccentricity, location × group, location × SF, eccentricity × group, eccentricity × SF, group × SF, location × eccentricity × group (*P = 0.006), location × eccentricity × SF all had significant effects on log CS (all **P < 0.001 except the explicitly stated).

*Post-hoc* analysis showed that, for all 19 SFs, the log CS of the myopia group was higher than that of the normal group at the superior (mean difference, 0.02; 95% CI, 0.01–0.20; *P = 0.02) and inferior (mean difference, 0.11; 95% CI, 0.02–0.21; *P = 0.01) visual field locations at 12°.

## Discussion

Our objective was to compare peripheral contrast sensitivity functions of myopes and emmetropes. We applied the qCSF method to assess CSF over a wide range of SF, eccentricities, and visual field locations. To our knowledge, this is the first study that quantified peripheral CSF differences between the two populations with the Bayesian adaptive qCSF method.

The study was designed to identify specific macular and peripheral locations where myopes and emmetropes may show differences in CSF. Consistent with Li et al. (2020), we found no contrast sensitivity difference between the emmetropes and myopes in a wide range of SFs in the fovea. However, we found increased contrast sensitivities in the myopia group at the para- (6°) and mid-peripheral (12°) eccentricities. The observation of increased contrast sensitivity at 6 and 12° of the superior and inferior visual fields in the myopia group suggests that peripheral contrast sensitivity may play some role in eye growth during emmetropization and contribute to myopia progression (Schmid and Wildsoe, 1997; Lai et al., 2008; Wolsley et al., 2008; Liu and Wildsoet, 2011). Interestingly, the superior, inferior, and nasal but not temporal visual field locations, mainly relevant to near work such as reading, exhibited enhanced contrast sensitivities. The findings may be related to cell distributions on the retina. The fovea consists of high-density cones which lead to high visual acuity. As eccentricity increases, cone density decreases and rod density increases, reaching equal densities at around 12° (Mohand-Said et al., 2001). Both rod- and cone-mediated responses drive spatial vision (Rieke et al., 2015). The observed enhancements of contrast sensitivity in myopia, mainly in the inferior visual field, may be related to the asymmetric distributions of retinal photoreceptor and ganglion cells along the horizontal and vertical directions. Multiple articles have demonstrated that visual functions differ in different parts of the visual field in myopia and the emmetropia, with visual acuity in the nasal and superior retinal regions being better than that in the temporal and inferior regions in myopia and emmetropia groups (Ehsaei et al., 2013). Wei et al. (2021) found significant correlation between myopia severity and motion detection thresholds in the nasal and superior visual field at 20°. Kuo et al. (2018) found significant correlations between Dmin task performance and myopia severity in the superior and temperal visual field. Ehsaei et al. (2013) suggested that the fall-off of visual performance with eccentricity was less pronounced in the horizontal than vertical meridians because of the higher density of retinal cells around the horizontal meridian. All these studies suggested that myopia development is closely related to changes of visual functions in the superior visual field. A complete understanding of the observed phenomenon, that is, whether it is due to redistribution of cones and rods on the peripheral retina or abnormal enhancement of visual processing in higher level visual pathway, needs further research. Future studies may apply physiological measures such as Electroencephalogram (EEG) and functional magnetic resonance imaging (fMRI) with peripheral retinal stimuli to evaluate higher level contributions.

It is well-known that emmetropization is an experience-dependent process. Normal visual experience is very important during the growth of the eyeball, optical defocusing can induce abnormal axial growth and lead to myopia (Wallman and Winawer, 2004; Schaeffel and Feldkaemper, 2015). Animal studies have shown that reducing the quality of visual input by covering the eye with a diffuser can lead to myopia, and the more severe the image degradation, the greater the degree of myopia (Wallman and Winawer, 2004; Morgan, 2010). Schmid and Wildsoe (1997) found that emmetropization was tuned by spatial frequency, and a mid-spatial frequency (0.86 cpd) stimulation could prevent deprivation myopia in young chicks. Hess et al. (2006) stimulated the eyes of young chicks with images at different spatial frequencies and found that low-frequency (0.084 cpd) spatial information stimulation is more likely to lead to accelerated myopia progression. Studies have also shown that emmetropia is maintained in the majority of animals presented with visual stimulation at 47.5% and higher contrasts, and high levels of myopia occur in the majority of animals presented with visual stimulations with contrast lower than 4.2%, which is at or below the behaviorally determined contrast threshold (Schmid et al., 2006). Moreover, the spatial frequency, contrast and other characteristics of the visual environment, especially in peripheral vision, have been recognized as visual signals that affect the occurrence and development of myopia (Smith et al., 2005). Animal experiments with peripheral form deprivation have demonstrated that the visual signals were integrated over restricted retinal regions and that peripheral image quality influenced local ocular growth in a manner that affects the overall axial length of the eye globe (Wallman et al., 1987; Wallman and Winawer, 2004). Because the fovea constitutes a small geographic area of the retina, the absolute number of retinal neurons in the fovea is much lower than that in the much larger periphery (Wallman and Winawer, 2004). Therefore the growth signals from the periphery may overshadow conflicting signals from the fovea and dominate overall axial growt (Smith et al., 2005). All these studies suggest that the spatial frequency, contrast and other characteristics of the visual environment may induce peripheral defocus (blur) and affect the emmetropization process, playing an important role in the occurrence and development of myopia. On the other hand, some studies have showed reduced contrast sensitivity as a consequence of peripheral blur (Venkataraman et al., 2015). More studies are necessary to determine whether the observed contrast sensitivity change is the cause or the consequence of myopia.

Although previous studies have investigated the difference between myopes and emmetropes in peripheral vision, they have focused on either visual acuity at fixed contrast levels or detection thresholds at a single spatial frequency, not the entire contrast sensitivity function. For example, Ehsaei et al. (2013) found decreased visual acuity at high contrast (100%) but comparable visual acuity at low contrast (14%) in myopic peripheral vision. Thorn et al. (2016) measured peripheral contrast detection thresholds binocularly at 8, 17, and 30° eccentricities and found no significant difference between myopes and emmetropes. Wei et al. (2021) found motion detection thresholds were negatively correlated with the degree of myopia for low spatial frequency targets at 20° eccentricity. In this study, we applied the Bayesian adaptive qCSF procedure and compared the contrast sensitivity functions of myopes and emmetropes in peripheral vision. CSF is a more comprehensive assessment of spatial vision.

We used soft contact lens for optical correction. Compared to spectacles, contact lens correction offers a wider field of view and may provide better corrected peripheral refraction. Liou and Chiu (2001) found that contact lens correction could reduce optical defocus and improve contrast sensitivity function in high spatial frequencies compared to spectacles. However, Ehsaei et al. (2011) did not find any significant difference in central and peripheral visual performance of myopic subjects under contact lens and spectacle lens correction, even with the consideration of spectacle magnification. Although we can’t completely rule out contributions of imperfect optical correction, the location-dependent increase of contrast sensitivity in myopia is unlikely caused by imperfect optical correction because it would decrease not increase contrast sensitivity.

A total of 52 eyes were included in this study, with large amount of data collection in each eye. One of the limitations of the study is the relatively small sample size, which may be the reason for the large variabilities in the myopia group. On the other hand, the SD of CSF metrics of the myopia group was within two times of that of the normal group at each eccentricity and each location; the variability was acceptable. Brown-Forsythe correction was used to correct the multivariate ANOVA results. In comparison, a total of 17 eyes were included in Wei et al.’s (2021) study and a total of 45 eyes were included in Thorn et al.’s (2016) study on peripheral visual functions. Both were small sample studies, perhaps due to the complexity of peripheral CSF measurements. Our observation of increased contrast sensitivities within the macula visual field in myopia was based on a sample size comparable to other peripheral myopia visual function studies (Timberlake et al., 1986; Maniglia et al., 2020). Importantly, our study was an exploratory experiment. Future studies with larger sample sizes are necessary to further evaluate our results.

In this study, we adopted the qCSF method and generated reliable measures of peripheral CSF, consistent with Rosen et al. (2014). The CSF was tested with a 10AFC digit identification task in central vision, but a 2-AFC grating orientation identification task in the periphery. First, the two procedures assessed performance at the same d’ level. Second, CSFs of the myopia and emmetropia groups were compared at the same locations, that is, the foveal CSF of the myopia group was compared with the foveal CSF of the emmetropia group, and peripheral CSFs of the myopia group were compared with the peripheral CSFs of the emmetropia group, and peripheral CSFs. There was no direct comparison between the foveal CSF from the 10AFC task and the peripheral CSFs from the 2-AFC task. Third, a multivariate ANOVA was performed to evaluate the effects of location and eccentricity on the CSF. We found no significant interaction between location and group. *Post-hoc* analysis showed that the difference was due to group not to location. Meanwhile, the 2-AFC grating identification task used to measure CSF still requires improvement. The testing time, although quite short, was still susceptible to visual fatigue. This limitation may be potentially remedied by the recently developed qVFM method which uses a 10-AFC letter identification paradigm to map contrast sensitivity across the entire visual field (Xu et al., 2019, 2020). Applications of the qVFM to clinical populations still require additional development and validation. A possible area of future research would be to apply peripheral CSF testing in myopia control clinical trials once we have finalized a more efficient paradigm.

## Conclusion

We observed enhanced contrast sensitivities within the superior and inferior quadrants of the parafoveal and mid-perifoveal zones of the retina in corrected myopia. These regions are relevant to near work, such as reading, which also generates high contrast visual signals to the retina. It is possible; therefore, that the increased contrast sensitivity in these peripheral regions of a myopic eye may serve as an external signal for further eye growth during emmetropization. However, On the other hand, some studies have showed reduced contrast sensitivity as a consequence of peripheral blur. We cannot completely rule out the possibility that increased CSF in the peripheral regions of a myopic eye may be the consequence but not the cause of myopia. More studies are necessary to determine whether the observed contrast sensitivity change is the cause or the consequence of myopia. These results may have strong implications for future research on myopia development from a functional visual-signal modulation perspective as well the relationship between the structure and functions of peripheral retina for constructing novel regulation models for myopia control.

## Data availability statement

The original contributions presented in this study are included in the article/Supplementary material, further inquiries can be directed to the corresponding authors.

## Author contributions

Z-LL, JL, ZX, and YJZ designed the research. ZC, JY, LF, and QY performed the research. ZX, YJZ, and ZC analyzed the data and drafted the manuscript. ZX, YJZ, ZC, FH, LC, YH, YSZ, YJ, Z-LL, and JL revised the manuscript. All authors commented on and edited the manuscript and approved the final version of the manuscript.
