# Supplementary material for: Assessing the contrast sensitivity function in myopic parafovea: A quick contrast sensitivity functions study
Source: Front Neurosci. 2022 Oct 6;16:971009. doi: 10.3389/fnins.2022.971009 (PMC9582454; doi:10.3389/fnins.2022.971009)
Supplement: Supplementary file 1 [file Data_Sheet_1.pdf]

Supplementary Material

Supplementary Table S1 Test spatial frequencies at four eccentricities

	**6°**	**12°**	**18°**	**24°**
1	0.70 cpd	0.70 cpd	0.70 cpd	0.70 cpd
2	0.84 cpd	0.82 cpd	0.81 cpd	0.80 cpd
3	1.02 cpd	0.96 cpd	0.94 cpd	0.91 cpd
4	1.22 cpd	1.13 cpd	1.08 cpd	1.04 cpd
5	1.47 cpd	1.33 cpd	1.25 cpd	1.19 cpd
6	1.78 cpd	1.56 cpd	1.44 cpd	1.35 cpd
7	2.14 cpd	1.83 cpd	1.67 cpd	1.54 cpd
8	2.58 cpd	2.15 cpd	1.93 cpd	1.76 cpd
9	3.11 cpd	2.52 cpd	2.23 cpd	2.01 cpd
10	3.74 cpd	2.96 cpd	2.58 cpd	2.29 cpd
11	4.51 cpd	3.47 cpd	2.98 cpd	2.61 cpd
12	5.43 cpd	4.07 cpd	3.45 cpd	2.98 cpd
13	6.54 cpd	4.78 cpd	3.98 cpd	3.40 cpd
14	7.88 cpd	5.61 cpd	4.60 cpd	3.88 cpd
15	9.49 cpd	6.59 cpd	5.32 cpd	4.43 cpd
16	11.44 cpd	7.73 cpd	6.15 cpd	5.05 cpd
17	13.78 cpd	9.07 cpd	7.11 cpd	5.76 cpd
18	16.60 cpd	10.65 cpd	8.22 cpd	6.50 cpd
19	20.00 cpd	12.50 cpd	9.50 cpd	7.50 cpd

Supplementary Table S2 Three-way between-subjects ANOVA results for AULCSF and cut-off SF

Results	Source	Sum of Squares	df	Mean Square	F	*p*-value
AULCSF	location	3.619	3	1.206	30.156	*<0.001***
	eccentricity	111.113	3	37.038	925.965	*<0.001***
	group	0.120	1	0.120	2.996	0.084
	location x eccentricity	6.848	9	0.761	19.023	*<0.001***
	location x group	0.111	3	0.037	0.914	0.434
	eccentricity x group	0.441	3	0.147	3.673	*0.012**
	location x eccentricity x group	0.111	9	0.012	0.307	0.973
	Error	33.279	832	0.040		
	Total	686.069	866			
Cut-off SF	location	127.982	3	42.661	7.499	*<0.001***
	eccentricity	8207.325	3	2735.775	480.897	*<0.001***
	group	4.397	1	4.397	0.773	0.380
	location x eccentricity	308.279	9	34.253	6.021	*<0.001***
	location x group	44.090	3	14.697	2.583	0.052
	eccentricity x group	17.055	3	5.685	0.999	0.393
	location x eccentricity x group	42.939	9	4.771	0.839	0.581
	Error	4317.879	759	5.689		
	Total	65025.175	793			

Abbreviations: df = degree of freedom

Supplementary Table S3 Four-way between-subjects ANOVA results for low- and intermediate-SF AULCSF

Source	Sum of Squares	df	Mean Square	F	*p-value*
Location	645.707	3	215.236	13.677	*<0.0001***
Eccentricity	17319.777	3	5773.259	366.851	*<0.0001***
Group	87.593	1	87.593	5.566	*0.018**
SF range	4312.039	1	4312.039	274.00 1	*<0.0001***
location x eccentricity	913.129	9	101.459	6.447	*<0.0001***
location x group	16.976	3	5.659	0.360	0.782
location x SF range	470.287	3	156.762	9.961	*<0.0001***
eccentricity x group	98.479	3	32.826	2.086	0.100
eccentricity x SF range	9910.938	3	3303.646	209.924	*<0.0001***
group x SF range	86.848	1	86.848	5.519	*0.019**
location x eccentricity x group	348.046	9	38.672	2.457	*0.009**
location x eccentricity x SF range	505.078	9	56.120	3.566	*<0.0001***
location x group x SF range	15.073	3	5.024	0.319	0.811
eccentricity x group x SF range	64.239	3	21.413	1.361	0.253
location x eccentricity x group x SF range	345.916	9	38.435	2.442	*0.009**
Error	25038.109	1591	15.737		
Total	103142.752	1655			

Abbreviations: df = degree of freedom

Supplementary Table S4 Two-way ANOVA results for high-SF AULCSF

Source	Sum of Squares	df	Mean Square	F	*p-value*
location	34.942	3	11.647	4.107	*0.007**
group	4.764	1	4.764	1.680	0.196
location x group	19.770	3	6.590	2.323	0.076
Error	567.256	200	2.836		
Total	1318.103	208			

Abbreviations: df = degree of freedom

Supplementary Table S5 Four-way between-subjects ANOVA results for log CS

Source	Sum of Squares	df	Mean Square	F	*p-value*
Location	103.676	3	34.559	415.374	*<0.001***
Eccentricity	706.543	3	235.514	2830.739	*<0.001***
Group	1.435	1	1.435	17.253	*<0.001***
SF	5685.140	18	315.841	3796.218	*<0.001***
location x eccentricity	297.417	9	33.046	397.197	*<0.001***
location x group	3.108	3	1.036	12.451	*<0.001***
location x SF	88.624	54	1.641	19.726	*<0.001***
eccentricity x group	7.861	3	2.620	31.495	*<0.001***
eccentricity x SF	182.499	54	3.380	40.621	*<0.001***
group x SF	4.465	18	0.248	2.982	*<0.001***
location x eccentricity x group	1.909	9	0.212	2.549	*0.006**
location x eccentricity x SF	129.367	162	0.799	9.598	*<0.001***
location x group x SF	1.097	54	0.020	0.244	1.000
eccentricity x group x SF	1.899	54	0.035	0.423	1.000
location x eccentricity x group x SF	5.846	162	0.036	0.434	1.000
Error	1258.300	15124	0.083		
Total	24937.112	15732			

Abbreviations: df = degree of freedom
